# Load-Sharing Model under Lindley Distribution and Its Parameter Estimation Using the Expectation-Maximization Algorithm

**DOI:** 10.3390/e22111329

**Published:** 2020-11-22

**Authors:** Chanseok Park, Min Wang, Refah Mohammed Alotaibi, Hoda Rezk

**Affiliations:** 1 Applied Statistics Laboratory, Department of Industrial Engineering, Pusan National University, Busan 46241, Korea; cpark2@gmail.com; 2 Department of Management Science and Statistics, The University of Texas at San Antonio, San Antonio, TX 78249, USA; min.wang3@utsa.edu; 3 Mathematical Sciences Department, College of Science, Princess Nourah Bint Abdulrahman University, P.O. Box 84428, Riyadh 11671, Saudi Arabia; hodaragab2009@yahoo.com; 4 Department of Statistics, Al-Azhar University, Cairo 11884, Egypt

**Keywords:** expectation-maximization algorithm, hypothetical latent random variable, Lindley distribution, maximum likelihood estimation, Newton–Raphson method

## Abstract

A load-sharing system is defined as a parallel system whose load will be redistributed to its surviving components as each of the components fails in the system. Our focus is on making statistical inference of the parameters associated with the lifetime distribution of each component in the system. In this paper, we introduce a methodology which integrates the conventional procedure under the assumption of the load-sharing system being made up of fundamental hypothetical latent random variables. We then develop an expectation maximization algorithm for performing the maximum likelihood estimation of the system with Lindley-distributed component lifetimes. We adopt several standard simulation techniques to compare the performance of the proposed methodology with the Newton–Raphson-type algorithm for the maximum likelihood estimate of the parameter. Numerical results indicate that the proposed method is more effective by consistently reaching a global maximum.

## 1. Introduction

We consider a load-sharing system which consists of several components associated in parallel. As each of the components fails one by one in the system, the surviving components will share the load with the remaining surviving components.

The load-sharing system has been studied since the 1940s [1]. Since then, it is widely used in many engineering applications [2,3,4]. The studies in [5,6] drew, in particular, load-sharing parameter inferences in a system with each component with a constant failure rate. Singh et al. [7] presented a more generalized load-sharing system under an assumption of a constant hazard at the beginning and a linearly increasing hazard rate after the failure of several components. Wang et al. [8] studied a more generalized load-sharing system with the work history or memory.

Park [9,10] considered the maximum likelihood estimate (MLE) of the parameters in the system, whose component lifetimes are distributed as exponential, Weibull, normal, and lognormal. Singh et al. [7] analyzed the discrete-type multi-component load-sharing parallel system and later on Deshpandé et al. [11] presented the nonparametric-type load-sharing system.

Singh and Gupta [12,13] and Singh et al. [14] investigated the load-sharing system model, in which the failure times of each component can be either constant or time-dependent. It deserves mentioning that Bayesian analyses of the load-sharing systems with several well-known distributions of lifetime components have also been investigated in the literature [15,16,17]. For instance, Xu et al. [15] studied a test scheme for reliability demonstration test on load-sharing systems with Weibull-distributed (including exponential) lifetime components. More recently, Singh and Goel [16] studied a multi-component parallel system model by assuming a load-sharing tendency on its components. Xu et al. [17] proposed the expectation-maximization (EM) algorithm for estimating the parameters in the load-sharing system with continuous degradation.

It is worthwhile to mention that based on accelerated testing models, the MLE method for the parameter estimation of the load-sharing systems under the classical latent variable approach is not well studied when the normal and lognormal are assumed to be the lifetime distribution of components. As mentioned in [5], a direct generalization of the load-sharing system with these lifetime distributions is usually problematic when making likelihood-based parametric inference, because of following reasons: (a) it may be impossible or extremely difficult to obtain explicit MLEs, (b) standard numerical methods such as the Newton–Raphson-type method can perform poorly when the dimension of parameters is high, or  (c) if the likelihood function is not sharp enough, then the standard numerical methods may fail to reach the global maximizer.

In this paper, we extend the load-sharing system with Lindley-distributed component lifetimes. Since the Lindley distribution was proposed by [18] as a counterexample of fiducial statistics, it has been widely used in reliability engineering due to its non-constant hazard rate in comparison to the exponential distribution. A natural question that arises is about making statistical inference of the parameters in this system. As mentioned above, the Newton–Raphson-type method can be sensitive to starting values so that it often fails to reach a correct solution especially for high-dimensional parameters. An alternative is to consider the EM algorithm particularly in engineering applications. For example, Albert and Baxter [19] derived the EM sequences with several causes, censoring, partial masking and right-censoring for the exponential model. Park [20] generalized their EM-type results in the system with different multi-modal strength distributions. Amari et al. [21] used the EM method to evaluate incomplete warranty data. Recently, Park [22] analyzed interval-data using the quantile implementation of the EM algorithm which improves the Monte Carlo EM (MCEM) algorithm’s convergence and stability properties and Ouyang [23] considered an interval-based model for analyzing incomplete data in a different viewpoint. These observations motivate us to develop an EM-type MLE for the parameters in the considered system to overcome the possible difficulties mentioned above when using the standard numerical methods.

The rest of the paper is as follows. In Section 2, we discuss the construction of the likelihood of the system with Lindley-distributed component lifetimes. In Section 3, we develop an EM algorithm for performing the MLE of the system under consideration. In Section 4, we conduct numerical studies to investigate the performance of the proposed EM-type MLE method, and some concluding remarks are given in Section 5.

## 2. Likelihood Construction

In this section, we briefly overview the construction of the likelihood function for the load-sharing system in Section 2.1 and then focus on the working likelihood in the load-sharing system with Lindley-distributed component lifetimes in Section 2.2.

### 2.1. Working Likelihood for the Load-Sharing Model

We here provide a brief description of the likelihood construction needed for a load-sharing framework and refer the interested readers to [9,10] for details on various types of load-sharing models. We assume that in a load-sharing parallel system consisting of *J* components, the failure rates of the remaining surviving components could change and the lifetimes of the surviving components at each failure step are independent and identically distributed (iid). However, since each of the components fails one by one (without being replaced or repaired) in the system, the parameter of the remaining surviving components should change from $\theta^{\left(0\right)}$ to $\theta^{\left(1\right)}$ after the occurrence of the first failure. Similarly, it also changes from $\theta^{\left(j-1\right)}$ to $\theta^{\left(j\right)}$ after the occurrence of the *j*-th failure. Let $Y^{\left(j\right)}$ be the lifetime between the failures of the *j*-th and (j+1)-st for j=0,1,…,J−1. We will observe the value of $Y^{\left(j\right)}$ after the failure of the *j*-th component. For notational simplicity, we assume that the component fails in the order of the *J*-th, the (J−1)-st, etc.

When analyzing the lifetime distribution in a load-sharing system, standard methods are developed based on the use of hypothetical latent random variables. After the occurrence of the *j*-th failure, there are J−j surviving components in the system. These surviving components are denoted by $U_{1}^{\left(j\right)},U_{2}^{\left(j\right)},\ldots ,U_{J-j}^{\left(j\right)}$, respectively. Then, we follow the traditional latent variable approach and model

(1) $$Y^{\left(j\right)}=\min\left\{U_{1}^{\left(j\right)},U_{2}^{\left(j\right)},\ldots ,U_{J-j}^{\left(j\right)}\right\}.$$

For a parametric model, each lifetime distribution usually includes the unknown parameters. We shall be particularly interested in obtaining the MLEs of the unknown parameters in the system. Given that it is often impossible to obtain the analytic-form MLE of the parameters, we often employ some reliable numerical procedures for obtaining the MLEs. Of particular note is that many lifetimes of the non-observed components can be considered to be right-censoring. For more details, the reader is directed to Equation (1.1) in [24], Section 5.3.1 in [25], Example 6.30 in [26], and the EM algorithm with right-censoring in Park [20]. This observation motivates us to adopt a similar method for constructing the likelihood of the load-sharing system as follows.

We assume that for a random sample of size *n*, the *i*-th measurement can be categorized as below

$$Y_{i}^{\left(j\right)}=\min\left\{U_{i,1}^{\left(j\right)},U_{i,2}^{\left(j\right)},\ldots ,U_{i,J-j}^{\left(j\right)}\right\}.$$

Let the realization of the random variables $Y_{i}^{\left(j\right)}$ be $y_{i}^{\left(j\right)}$. By using the formulation in [9,10], we can construct the observed likelihood function from the *n* observations as follows

$$L\left(\Theta \right)\propto \prod_{i=1}^{n}\prod_{j=0}^{J-1}f_{Y}^{\left(j\right)}\left(y_{i}^{\left(j\right)}\right),$$

where $\Theta =(\theta^{\left(0\right)},\theta^{\left(1\right)},\ldots ,\theta^{\left(J-1\right)})$,

$$f_{Y}^{\left(j\right)}\left(y\right)=\prod_{\ell =1}^{J-j}S_{U_{\ell }}^{\left(j\right)}\left(y\right)\times \sum_{\ell =1}^{J-j}h_{U_{\ell }}^{\left(j\right)}\left(y\right)\;\;\mathrm{and}\;\;h_{U_{\ell }}^{\left(j\right)}\left(y\right)=\frac{f_{U_{\ell }}^{\left(j\right)}\left(y\right)}{S_{U_{\ell }}^{\left(j\right)}\left(y\right)}.$$

Under the equal load-sharing rule, we consider the parameter estimates for the system with Lindley-distributed component lifetimes. For the notion of completeness, we here review the likelihood construction provided in [10]. We assume that for a specific value of *j*, $U_{\ell }^{\left(j\right)}$ are *iid* for ℓ=1,2,…,J−j. The cumulative distribution function (cdf) of $Y^{\left(j\right)}$ can then be defined as

(2) $$P\left[Y^{\left(j\right)}\leq y\right]=1-P\left[Y^{\left(j\right)}>y\right]=1-S_{U}^{\left(j\right)}{\left(y\right)}^{J-j},$$

where $S_{U}^{\left(j\right)}\left(\cdot \right)$ is the survival function of $U_{\ell }^{\left(j\right)}$ with the parameter $\theta^{\left(j\right)}$, where j=0,1,…,J−1. Note that we omitted the index *ℓ* for $S_{U}^{\left(j\right)}\left(\cdot \right)$ since $U_{\ell }^{\left(j\right)}$ are *iid* for any *ℓ*.

Let $h_{U}^{\left(j\right)}\left(y\right)=f_{U}^{\left(j\right)}\left(y\right)/S_{U}^{\left(j\right)}\left(y\right)$ be the hazard function. The probability density function (pdf) of $Y^{\left(j\right)}$ can be easily obtained by differentiating Equation (2), and it is given by

(3) $$f_{Y}^{\left(j\right)}\left(y\right)=\left(J-j\right)h_{U}^{\left(j\right)}\left(y\right)S_{U}^{\left(j\right)}{\left(y\right)}^{J-j},$$

Then the likelihood and log-likelihood functions for the *n* observations are, respectively, given by

(4) $$\begin{array}{c} L\left(\Theta \right)\propto \prod_{i=1}^{n}\prod_{j=0}^{J-1}f_{Y}^{\left(j\right)}\left(y_{i}^{\left(j\right)}\right) \end{array}$$

and

(5) $$\begin{array}{c} \ell \left(\Theta \right)\propto \sum_{i=1}^{n}\sum_{j=0}^{J-1}\log f_{Y}^{\left(j\right)}\left(y_{i}^{\left(j\right)}\right). \end{array}$$

### 2.2. Likelihood Construction with the Lindley Distribution

We focus on the load-sharing system with Lindley-distributed component lifetimes. The pdf and cdf of the Lindley distribution with the parameter θ>0 are given by

(6) $$f\left(x\right)=\frac{\theta^{2}\left(1+x\right)e^{-\theta x}}{\left(\theta +1\right)}\;\;\mathrm{and}\;\;F\left(x\right)=1-\frac{\left(\theta +1+\theta x\right)e^{-\theta x}}{\theta +1},$$

respectively, for x>0. Then the survival and hazard functions of the Lindley distribution are given by S(x)=1−F(x) and h(x)=f(x)/S(x), respectively. Based on the Lindley distribution above, we have the survival and hazard functions of $U_{\ell }^{\left(j\right)}$

(7) $$S_{U}^{\left(j\right)}\left(y\right)=\frac{\left(\theta^{\left(j\right)}+1+\theta^{\left(j\right)}y\right)e^{-\theta^{\left(j\right)}y}}{\theta^{\left(j\right)}+1}\;\mathrm{and}\;h_{U}^{\left(j\right)}\left(y\right)=\frac{{\theta^{\left(j\right)}}^{2}\left(1+y\right)}{\theta^{\left(j\right)}+1+\theta^{\left(j\right)}y}.$$

Thus, using Equations (3) and (7), it follows that

(8) $$f_{Y}^{\left(j\right)}\left(y\right)=\left(J-j\right)\frac{{\theta^{\left(j\right)}}^{2}\left(1+y\right)}{\theta^{\left(j\right)}+1+\theta^{\left(j\right)}y}{\left[\frac{\left(\theta^{\left(j\right)}+1+\theta^{\left(j\right)}y\right)e^{-\theta^{\left(j\right)}y}}{\theta^{\left(j\right)}+1}\right]}^{J-j}.$$

Substitution of Equations (8) into (5) leads to

(9) $$\begin{array}{cc} \ell (\Theta )\;\propto \;\; & 2n\sum_{j=0}^{J-1}\log\theta^{\left(j\right)}+\sum_{i=1}^{n}\sum_{j=0}^{J-1}\log\left(1+y_{i}^{\left(j\right)}\right)+\sum_{i=1}^{n}\sum_{j=0}^{J-1}\left(J-j-1\right)\log\left(\theta^{\left(j\right)}+1+\theta^{\left(j\right)}y_{i}^{\left(j\right)}\right)\\ \; & -n\sum_{j=0}^{J-1}\left(J-j\right)\log\left(\theta^{\left(j\right)}+1\right)-\sum_{i=1}^{n}\sum_{j=0}^{J-1}\left(J-j\right)\theta^{\left(j\right)}y_{i}^{\left(j\right)}. \end{array}$$

To the best of our knowledge, it is actually impossible to derive an analytic-expression of the MLE of the likelihood function in Equation (9). Thus, appropriate numerical procedures are usually required to obtain the MLE under this scenario, whereas, as afore-mentioned, the conventional Newton–Raphson-type method is very sensitive to starting values and it could often fail to reach a correct solution especially for high-dimensional parameters. Given that the likelihood in Equation (9) can be easily over-parameterized, we recommend the use of an EM algorithm to overcome these difficulties in maximizing the likelihood in Equation (4) discussed as follows.

## 3. The Proposed EM Algorithm

We first briefly discuss how to implement an EM algorithm based on the complete-data likelihood function in Section 3.1 and develop the EM-type MLEs for the load-sharing system with Lindley-distributed component lifetimes in Section 3.2.

### 3.1. An EM Algorithm with the Load-Sharing Model

The EM algorithm [24] is a numerical iterative algorithm to obtain the MLE of parametric models. The EM algorithm consists of two steps which are (i) an expectation step (E-step) and (ii) a maximization step (M-step). It is often employed to solve a difficult or complex likelihood problem to obtain the MLE by iterating two easier steps above. In the E-step, we calculate the expectation of the log-likelihood function under the incomplete data given the observed data. In the M-step, we calculate the maximizer of the expected log-likelihood. Although this algorithm may be slower than the gradient-based methods such as Newton–Raphson, it is very stable. For the sake of completeness, we briefly discuss the EM algorithm when missing values such as censored or masked data are considered. For more details on the term *missing values* in terms of data coarsening, the reader is referred to the references [26,27,28].

Let θ denote the set of unknown parameters. We assume that the complete data $x=(x_{1},x_{2},\ldots ,x_{n})$ can be reorganized as x=(y,z), where $y=(y_{1},y_{2},\ldots ,y_{m})$ represents the *fully observed* part and $z=(z_{m+1},z_{m+2},\ldots ,z_{n})$ stands for the *missing* part (right-censored in the load-sharing) [24]. Then the likelihood function becomes

$$L_{c}\left(\theta |y,z\right)=\prod_{i=1}^{m}f\left(y_{i}|\theta \right)\prod_{i=m+1}^{n}f\left(z_{i}|\theta \right)$$

which can also be rewritten as

$$L_{c}\left(\theta |x\right)=\prod_{i=1}^{n}f\left(x_{i}\right).$$

For more details on the above, see Section 5.3.1 in [25] and Section 6.3.3 in [26]. Let $\theta_{s}$ be the estimate at the *s*-th step of the algorithm and define

$$E\left[\log L_{c}\left(\theta |y,z\right)\right]=\int \log L_{c}\left(\theta |y,z\right)\;f\left(z|y,\theta_{s}\right)\;dz.$$

The two distinct steps of the EM algorithm can be summarized as follows:

- E-step:
  $\mathrm{Calculate}:\;Q\left(\theta |\theta_{s}\right)=E\left[\log L_{c}\left(\theta |y,z\right)\right].$
- M-step:
  $\mathrm{Obtain}:\;\;\theta_{s+1}=\arg\max_{\theta }Q\left(\theta |\theta_{s}\right).$

As yet, it is not clear how to implement the EM algorithm for performing the MLE of the considered load-sharing system. Given that the lifetime between the failures of the *j*-th and (j+1)-st is observed from the *minimum lifetime* among the remaining J−j components, we could view this load-sharing system in the setting of competing risks [29]. Consequently, after the occurrence of the *j*-th failure, let $U_{1}^{\left(j\right)},U_{2}^{\left(j\right)},\ldots ,U_{J-j}^{\left(j\right)}$ be the lifetimes of the remaining J−j surviving components, in which these components will compete with each other for the next failure. Also, given that the lifetime between the failures of the *j*-th and (j+1)-st are partially observed with the unknown component, we may view the system as masking [30]. Then, it is equivalent to assuming that causes (failed components) are completely masked or missing [20,31]. Thus, analogous to his approach to complete masking, we can easily construct the likelihood function of the system for implementing the EM algorithm as follows.

First, we use an indicator function and a variable to express the cause of failure of the component: let I{C∣D} be the indicator function of two event *C* and *D* and $\Delta_{i}^{\left(j\right)}$ be an indicator variable such that $\Delta_{i}^{\left(j\right)}=\ell$ indicating that the failure of the *ℓ*-th component. Then we define

(10) $$B_{i}^{\left(j\right)}\left(\ell \right)=I\left\{\Delta_{i}^{\left(j\right)}=\ell \;|\;y_{i}^{\left(j\right)}\right\}\;\;\mathrm{for}\;\ell =1,2,\ldots ,J-j.$$

Since $B_{i}^{\left(j\right)}\left(\ell \right)$ are the indicator functions of the events for ℓ=1,2,…,J−j, they have an *iid* Bernoulli distribution with the probability $P\left[B_{i}^{\left(j\right)}\left(\ell \right)=1\right]$, which is obtained as

$$P\left[B_{i}^{\left(j\right)}\left(\ell \right)=1\right]=P\left[\Delta_{i}^{\left(j\right)}=\ell \;|\;y_{i}^{\left(j\right)}\right],$$

for ℓ=1,2,…,J−j. Analogous to the approach in Section 8.2.2 of [32], the Bernoulli probability is obtained by

(11) $$P\left[B_{i}^{\left(j\right)}\left(\ell \right)=1\right]=\frac{1}{J-j},$$

where i=1,2,…,n and ℓ=1,2,…,J−j. For the detailed explicit derivation of Equation (11), one can refer to Appendix A in [10].

Next, we incorporate the above Bernoulli random variables and construct the complete-data likelihood function as follows. To be more specific, by using Equation (4), the likelihood function can be constructed as $L\left(\Theta \right)\propto \prod_{i=1}^{n}\prod_{j=0}^{J-1}f_{Y}^{\left(j\right)}\left(y_{i}^{\left(j\right)}\right),$ which can be rewritten as $L\left(\Theta \right)\propto \prod_{j=0}^{J-1}L\left(\theta^{\left(j\right)}\right)$, where $L\left(\theta^{\left(j\right)}\right)=\prod_{i=1}^{n}f_{Y}^{\left(j\right)}\left(y_{i}^{\left(j\right)}\right)$. Using the above Bernoulli random variables in Equation (10), we can rewrite $f_{Y}^{\left(j\right)}\left(y_{i}^{\left(j\right)}\right)$ for the complete-data likelihood as below

(12) $$f_{Y}^{\left(j\right)}\left(y_{i}^{\left(j\right)}\right)=\prod_{\ell =1}^{J-j}\left\{{f_{U}^{\left(j\right)}\left(y_{i}^{\left(j\right)}\right)}^{B_{i}^{\left(j\right)}\left(\ell \right)}\times {S_{U}^{\left(j\right)}\left(y_{i}^{\left(j\right)}\right)}^{1-B_{i}^{\left(j\right)}\left(\ell \right)}\right\},$$

which results in the following likelihood function

$$L\left(\theta^{\left(j\right)}\right)=\prod_{i=1}^{n}\prod_{\ell =1}^{J-j}\left\{{f_{U}^{\left(j\right)}\left(y_{i}^{\left(j\right)}\right)}^{B_{i}^{\left(j\right)}\left(\ell \right)}\times {S_{U}^{\left(j\right)}\left(y_{i}^{\left(j\right)}\right)}^{1-B_{i}^{\left(j\right)}\left(\ell \right)}\right\}.$$

For convenience, we rewrite the above likelihood function as

(13) $$L\left(\theta^{\left(j\right)}\right)=\prod_{i=1}^{n}L\left(\theta^{\left(j\right)}|y_{i}^{\left(j\right)}\right),$$

where $L(\theta^{\left(j\right)}|y_{i}^{\left(j\right)})$ stands for the likelihood function of the *i*-th system given by

(14) $$L\left(\theta^{\left(j\right)}|y_{i}^{\left(j\right)}\right)=\prod_{\ell =1}^{J-j}\left\{{f_{U}^{\left(j\right)}\left(y_{i}^{\left(j\right)}\right)}^{B_{i}^{\left(j\right)}\left(\ell \right)}\times {S_{U}^{\left(j\right)}\left(y_{i}^{\left(j\right)}\right)}^{1-B_{i}^{\left(j\right)}\left(\ell \right)}\right\}.$$

We observe that the complete-data likelihood function in (13) can now be constructed based on the Bernoulli random variable $B_{i}^{\left(j\right)}\left(\ell \right)$ in (10), whereas it still seems difficult to directly derive an explicit Q(·) function from Equation (13) in the E-step. We may overcome this difficulty by viewing the censored data as missing. This idea could help us avoid the use of the survival function, since in many practical cases, it is easier to deal with a density than a survival function. We thus provide a method for constructing the complete-data likelihood based on a density rather than a survival function, which could ease the difficulty in calculating the E-step. It should be noted that $S_{U}^{\left(j\right)}\left(y_{i}^{\left(j\right)}\right)=\int_{y_{i}^{\left(j\right)}}^{\infty }f_{U}^{\left(j\right)}\left(z\right)dz$. Thus, by introducing the random variable $Z_{i}$ for $Z_{i}>y_{i}^{\left(j\right)}$, we can replace $S_{U}^{\left(j\right)}\left(y_{i}^{\left(j\right)}\right)$ in Equation (14) with $f_{U}^{\left(j\right)}\left(Z_{i}^{\left(j\right)}\right)$ in the E-step. That is, $L(\theta^{\left(j\right)}|y_{i}^{\left(j\right)})$ can be re-expressed as

(15) $$L_{c}\left(\theta^{\left(j\right)}|y_{i}^{\left(j\right)},Z_{i}^{\left(j\right)}\right)=\prod_{\ell =1}^{J-j}\left\{{f_{U}^{\left(j\right)}\left(y_{i}^{\left(j\right)}\right)}^{B_{i}^{\left(j\right)}\left(\ell \right)}\times {f_{U}^{\left(j\right)}\left(Z_{i}^{\left(j\right)}\right)}^{1-B_{i}^{\left(j\right)}\left(\ell \right)}\right\},$$

where the pdf of $Z_{i}$ is defined as

(16) $$f_{Z}^{\left(j\right)}\left(t\right)=\frac{f_{U}^{\left(j\right)}\left(t\right)}{1-F_{U}^{\left(j\right)}\left(y_{i}^{\left(j\right)}\right)}\;\;\;\mathrm{for}\;\;t>y_{i}^{\left(j\right)}.$$

This technique is widely used for simplifying the calculation of the E-step. The reader is referred to Equation (1.1) in [24], Section 5.3.1 in [25], Example 6.30 in [26], and Park [20] for details. Now, using Equation (15), we can obtain an explicit closed-form Q(·) function which will be shown in Equation (21). This brings great simplicity for finding the MLE in the M-step.

Since $P[B_{i}^{\left(j\right)}\left(\ell \right)=1]=1/\left(J-j\right)$ for all *i* and *ℓ* as shown in Equation (11), we just write $B^{\left(j\right)}$ by ignoring *i* and *ℓ* for notational simplicity. Then the likelihood function of the complete-data in Equation (15) can be written as

(17) $$\begin{array}{cc} L_{c}^{*}\left(\theta^{\left(j\right)}\right)\; & =\prod_{i=1}^{n}L_{c}\left(\theta^{\left(j\right)}|y_{i}^{\left(j\right)},Z_{i}^{\left(j\right)}\right)\\ \; & =\prod_{i=1}^{n}\prod_{\ell =1}^{J-j}\left\{{f_{U}^{\left(j\right)}\left(y_{i}^{\left(j\right)}\right)}^{B^{\left(j\right)}}\;\;\;\times {f_{U}^{\left(j\right)}\left(Z_{i}^{\left(j\right)}\right)}^{1-B^{\left(j\right)}}\right\}\\ \; & =\prod_{i=1}^{n}{\left\{{f_{U}^{\left(j\right)}\left(y_{i}^{\left(j\right)}\right)}^{B^{\left(j\right)}}\;\;\;\times {f_{U}^{\left(j\right)}\left(Z_{i}^{\left(j\right)}\right)}^{1-B^{\left(j\right)}}\right\}}^{J-j}. \end{array}$$

In what follows, using Equation (17), we implement the EM algorithm for finding the MLE of the parameters in the load-sharing system with Lindley-distributed component lifetimes.

### 3.2. The EM-Type Maximum Likelihood Estimates with the Lindley Distribution

Since the lifetime of the components follows the Lindley distribution with the pdf in Equation (6). Then, the pdf of $U_{\ell }^{\left(j\right)}$ is defined as

$$f_{U}^{\left(j\right)}\left(t\right)=\frac{{\theta^{\left(j\right)}}^{2}}{\theta^{\left(j\right)}+1}\left(1+t\right)e^{-\theta^{\left(j\right)}t}.$$

We substitute this function into Equation (17) and take the logarithm of $L_{c}^{*}\left(\theta^{\left(j\right)}\right)$, which leads to the complete-data log-likelihood function of $\theta^{\left(j\right)}$ given by

(18) $$\begin{array}{cc} \log L_{c}^{*}\left(\theta^{\left(j\right)}\right)\; & =\left(J-j\right)[2n\log\theta^{\left(j\right)}-n\log\left(\theta^{\left(j\right)}+1\right)+B^{\left(j\right)}\sum_{i=1}^{n}\log\left(1+y_{i}^{\left(j\right)}\right)-B^{\left(j\right)}\theta^{\left(j\right)}\sum_{i=1}^{n}y_{i}^{\left(j\right)}\\ \; & \;\;+\left(1-B^{\left(j\right)}\right)\sum_{i=1}^{n}\log\left(1+Z_{i}^{\left(j\right)}\right)-\left(1-B^{\left(j\right)}\right)\theta^{\left(j\right)}\sum_{i=1}^{n}Z_{i}^{\left(j\right)}]\\ \; & =\left(J-j\right)\left[2n\log\theta^{\left(j\right)}-n\log\left(\theta^{\left(j\right)}+1\right)-B^{\left(j\right)}\theta^{\left(j\right)}\sum_{i=1}^{n}y_{i}^{\left(j\right)}-\left(1-B^{\left(j\right)}\right)\theta^{\left(j\right)}\sum_{i=1}^{n}Z_{i}^{\left(j\right)}\right]+C, \end{array}$$

where

$$C=\left(J-j\right)\left[B^{\left(j\right)}\sum_{i=1}^{n}\log\left(1+y_{i}^{\left(j\right)}\right)+\left(1-B^{\left(j\right)}\right)\sum_{i=1}^{n}\log\left(1+Z_{i}^{\left(j\right)}\right)\right].$$

It should be noted that *C* does not include $\theta^{\left(j\right)}$. Thus, we can treat it as a constant in the M-step, where Q(·) function is differentiated with respect to $\theta^{\left(j\right)}$.

Let $\theta_{s}^{\left(j\right)}$ be the estimate of $\theta^{\left(j\right)}$ at the *s*-th EM sequences. We can summarize the proposed EM as follows.

- E-step:
  Using Equations (11) and (18), we obtain
  (19) $$\begin{array}{cc} Q(\theta^{\left(j\right)}|\theta_{s}^{\left(j\right)})\; & =E[\log L_{c}^{*}\left(\theta^{\left(j\right)}\right)|\theta_{s}^{\left(j\right)}]\\ \; & =\left(J-j\right)[2n\log\theta^{\left(j\right)}-n\log\left(\theta^{\left(j\right)}+1\right)-\frac{1}{J-j}\theta^{\left(j\right)}\sum_{i=1}^{n}y_{i}^{\left(j\right)}\\ \; & \;-\left(1-\frac{1}{J-j}\right)\theta^{\left(j\right)}\sum_{i=1}^{n}E\left[Z_{i}^{\left(j\right)}|\theta_{s}^{\left(j\right)}\right]]+D. \end{array}$$
  where $D=E[C|\theta_{s}^{\left(j\right)}]$. Since the lifetime of each component follows the Lindley distribution, we observe from Equation (16) that the pdf of $Z_{i}^{\left(j\right)}|\theta_{s}^{\left(j\right)}$ can be written as
  $$f_{Z}^{\left(j\right)}\left(z\right)=\frac{{\theta_{s}^{\left(j\right)}}^{2}}{\theta_{s}^{\left(j\right)}+\theta_{s}^{\left(j\right)}y_{i}^{\left(j\right)}+1}\left(1+z\right)e^{-\theta_{s}^{\left(j\right)}\left(z-y_{i}^{\left(j\right)}\right)},$$
  where $z>y_{i}^{\left(j\right)}$. Then we obtain
  (20) $$E\left[Z_{i}^{\left(j\right)}|\theta_{s}^{\left(j\right)}\right]=\int_{y_{i}^{\left(j\right)}}^{\infty }zf_{Z}^{\left(j\right)}\left(z\right)dz=y_{i}^{\left(j\right)}+\frac{\theta_{s}^{\left(j\right)}+\theta_{s}^{\left(j\right)}y_{i}^{\left(j\right)}+2}{\theta_{s}^{\left(j\right)}\left(\theta_{s}^{\left(j\right)}+\theta_{s}^{\left(j\right)}y_{i}^{\left(j\right)}+1\right)}.$$
  It is immediate upon substituting Equation (20) into (19) that we obtain
  (21) $$Q\left(\theta^{\left(j\right)}|\theta_{s}^{\left(j\right)}\right)=n\left(J-j\right)\left[2\log\theta^{\left(j\right)}-\log\left(\theta^{\left(j\right)}+1\right)-\theta^{\left(j\right)}{\bar{w}}_{s}^{\left(j\right)}\right]+D,$$
  where
  $${\bar{w}}_{s}^{\left(j\right)}=\frac{1}{n}\sum_{i=1}^{n}\left[y_{i}^{\left(j\right)}+\left(\frac{J-j-1}{J-j}\right)\frac{\theta_{s}^{\left(j\right)}+\theta_{s}^{\left(j\right)}y_{i}^{\left(j\right)}+2}{\theta_{s}^{\left(j\right)}\left(\theta_{s}^{\left(j\right)}+\theta_{s}^{\left(j\right)}y_{i}^{\left(j\right)}+1\right)}\right].$$
- M-step:
  We differentiate $Q(\theta^{\left(j\right)}|\theta_{s}^{\left(j\right)})$ with respect to $\theta^{\left(j\right)}$ and obtain
  $$\frac{\partial Q(\theta^{\left(j\right)}|\theta_{s}^{\left(j\right)})}{\partial \theta^{\left(j\right)}}=n\left(J-j\right)\left[\frac{2}{\theta^{\left(j\right)}}-\frac{1}{\theta^{\left(j\right)}+1}-{\bar{w}}_{s}^{\left(j\right)}\right].$$
  Finally, we set the above equation to be zero in order to solve for $\theta^{\left(j\right)}$ and obtain the (s+1)-st EM sequences, denoted by $\theta_{s+1}^{\left(j\right)}=\theta^{\left(j\right)}$, such that
  (22) $$\theta_{s+1}^{\left(j\right)}=\frac{-\left({\bar{w}}_{s}^{\left(j\right)}-1\right)+\sqrt{{\left({\bar{w}}_{s}^{\left(j\right)}-1\right)}^{2}+8{\bar{w}}_{s}^{\left(j\right)}}}{2{\bar{w}}_{s}^{\left(j\right)}}.$$

We can easily obtain the MLE of the parameter in the system based on the above EM sequences. In addition, to make our procedure be accessible for practitioners, we provide the function written in R language [33] in Appendix A.

## 4. Numerical Study

### 4.1. Real Data Analysis

We consider the real data set analyzed in [12,34] to illustrate the usefulness of the proposed procedure. We refer the readers to [12,34] for more details on the description of the data. Singh and Gupta [12] used the load-sharing model with the underlying Lindley distribution and estimated ${\hat{\theta }}^{\left(0\right)}=0.036246642$, ${\hat{\theta }}^{\left(1\right)}=0.040587738$, and  ${\hat{\theta }}^{\left(2\right)}=0.069157598$ based on the Newton–Raphson-type method. The corresponding log-likelihood and likelihood values are −340.2890 and $1.638193\times 10^{-148}$, respectively.

We reanalyze the above real data with the proposed EM algorithm in Section 3. The R function for this analysis (Lindley.LS.EM) and the real data set are provided in Appendix A. Using the Lindley.LS.EM function, we obtained ${\hat{\theta }}^{\left(0\right)}=0.03624714$, ${\hat{\theta }}^{\left(1\right)}=0.04104211$, and  ${\hat{\theta }}^{\left(2\right)}=0.06915810$. The corresponding log-likelihood and likelihood values are −340.079 and $2.021003\times 10^{-148}$, respectively. Comparing the two results, we observe that the estimates using the EM algorithm have a greater likelihood value, which clearly implies that it is more effective than the Newton–Raphson-type method by Singh and Gupta [12].

### 4.2. Sensitivity of Parameter Estimation Due to Starting Values

We compare the sensitivity of starting values to the Newton–Raphson-type method and the EM algorithm. To this end, we generated ten lifetimes (n=10) from a five-component parallel system with the parameter values of $\theta^{\left(0\right)}=0.01$, $\theta^{\left(1\right)}=0.02$, $\theta^{\left(2\right)}=0.03$, $\theta^{\left(3\right)}=0.04$, and  $\theta^{\left(4\right)}=0.05$. Please note that Ghitanya et al. [35] used the mixture model to generate the Lindley random sample, whereas the sample can be directly obtained by solving F(x)=p for *x* where *p* is a uniform random number in (0,1). In Appendix B, we provided the quantile function of the Lindley distribution by solving F(x)=p for *x*. We provided the generated data in Table 1.

We first need to choose starting values for using both methods to obtain the estimates. We selected starting values at random and obtained the estimates based on the Newton–Raphson-type method and the EM algorithm. For the case of the proposed EM method, we obtained ${\hat{\theta }}^{\left(0\right)}=0.00837$, ${\hat{\theta }}^{\left(1\right)}=0.01994$, ${\hat{\theta }}^{\left(2\right)}=0.03099$, ${\hat{\theta }}^{\left(3\right)}=0.03908$ and ${\hat{\theta }}^{\left(4\right)}=0.05589$ along with the corresponding log-likelihood value $\ell (\hat{\Theta })=-223.63$ where $\hat{\Theta }=\left({\hat{\theta }}^{\left(0\right)},{\hat{\theta }}^{\left(1\right)},{\hat{\theta }}^{\left(2\right)},{\hat{\theta }}^{\left(3\right)},{\hat{\theta }}^{\left(4\right)}\right)$ regardless of starting values. On the other hand, for the case of the Newton–Raphson-type method, the parameter estimates are dependent on the choice of starting values. In Table 2, we reported only some non-convergent cases for brevity. These results clearly show that the Newton–Raphson-type method is sensitive to the choice of starting values.

We investigate the sensitivity of the estimates more thoroughly as follows.

**Figure 1:**
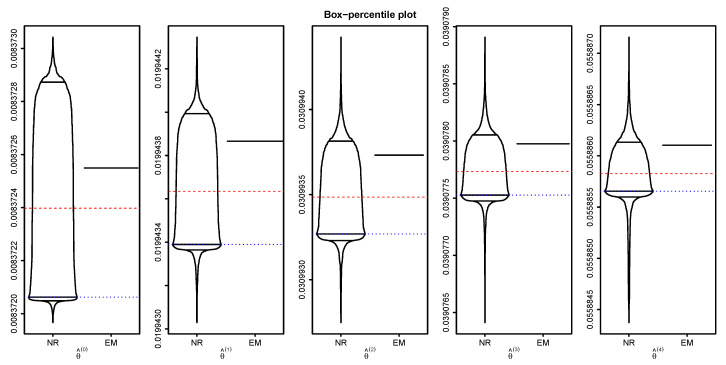
Box-percentile plots of the estimates based on the Newton–Raphson-type method (NR) and the EM algorithm.

Here, we choose starting values by randomly selecting a value in (0,1) and then estimate the five parameters using both methods. We repeated this procedure 1000 times, which results in 1000 sets of parameter estimates. We draw the box-percentile plots [36] of the estimates under each method in Figure 1. It is easily seen from the figure that the Newton–Raphson-type method shows the wide spread of the estimates indicating that it is sensitive to starting values, while the EM algorithm does not show any spread of the estimates which clearly shows that it is not sensitive to starting values at all. In the figure, we also added the mean (red dashed line) and median (blue dotted line) of the estimates using the Newton–Raphson-type method which indicate that they tend to underestimate the true MLE but their distributions are skewed to the right.

It deserves mentioning that we have more extensive results, but due to the space limitations, we briefly report some important findings. As expected, with an increasing number of parameters, the Newton–Raphson-type method becomes more unstable, and in some cases, it even fails to provide reasonable results. On the other hand, the EM algorithm always provides reliable estimates. It is of interest to note that for a small number of the parameters (e.g., five or smaller), the Newton–Raphson-type method is not so bad. In summary, when the number of the parameter is more than five, we highly recommend the use of the EM algorithm.

## 5. Conclusions

In this paper, we have introduced a methodology by integrating the conventional procedure under the assumption of the load-sharing system being made up of fundamental hypothetical latent random variables. In addition, we have investigated the problem of estimating the parameter of a load-sharing system with Lindley-distributed component lifetimes. To our knowledge, it is not possible to obtain a closed-form MLE of the parameter under this system. We thus develop an EM algorithm for finding the MLE of the load-sharing system under consideration. The EM algorithm is not only easily implemented by practitioners with the R programs in Appendix A, but also can alleviate some potential issues faced by iterative numerical methods such as the Newton–Raphson-type method.

We have conducted extensive simulations to compare the performance of the EM algorithm with the Newton–Raphson-type method. Numerical results indicated that the Newton–Raphson-type method often fails to converge and is very dependent on the starting values. Also, as the number of load-sharing parameters increases, it becomes increasingly ineffective because the chance of poor convergence becomes greater. Consequently, we do not recommend the use of this method for parameter estimation of the described load-sharing system. Instead, we have a preference for the proposed EM algorithm, because it consistently offers reliable results, even as the number of parameters becomes larger.

## Figures and Tables

**Table 1:** Lifetimes for load-sharing sample of size n=10.

$Y_{i}^{\left(0\right)}$	83.10	74.91	164.79	60.12	81.91	132.68	16.36	54.71	130.95	39.10
$Y_{i}^{\left(1\right)}$	59.25	53.01	60.31	46.38	20.69	25.53	56.11	28.99	23.04	32.21
$Y_{i}^{\left(2\right)}$	30.13	12.06	32.91	47.96	21.64	4.80	19.79	17.11	49.43	66.28
$Y_{i}^{\left(3\right)}$	47.29	10.45	6.43	17.80	67.01	37.41	41.89	20.17	21.62	41.60
$Y_{i}^{\left(4\right)}$	46.24	39.60	17.67	18.72	50.69	29.66	14.16	52.53	10.02	69.11

**Table 2:** Parameter estimates with corresponding log-likelihood values when the Newton–Raphson-type method is used.

Starting Values					Parameter Estimates					Log-Likelihood
$\theta^{\left(0\right)}$	$\theta^{\left(1\right)}$	$\theta^{\left(2\right)}$	$\theta^{\left(3\right)}$	$\theta^{\left(4\right)}$	${\hat{\theta }}^{\left(0\right)}$	${\hat{\theta }}^{\left(1\right)}$	${\hat{\theta }}^{\left(2\right)}$	${\hat{\theta }}^{\left(3\right)}$	${\hat{\theta }}^{\left(4\right)}$	$\ell (\hat{\Theta })$
0.004	1.25	2.98	3.89	4.24	0.00822	0.02001	0.03104	0.03876	0.05576	−223.64
0.028	1.31	2.04	3.18	4.18	0.01059	0.01959	0.03047	0.03764	0.05875	−224.50
0.021	1.18	2.69	3.38	4.77	0.01208	0.02297	0.02731	0.03693	0.05474	−226.24
0.028	1.97	2.66	3.67	4.21	0.04123	0.02421	0.06983	0.02912	0.05308	−307.76
0.026	1.72	2.91	3.95	4.07	0.04452	0.01781	0.03617	0.02440	0.07059	−308.38
0.002	1.90	2.75	3.24	4.12	0.05032	0.01995	0.03164	0.04057	0.05700	−323.65
0.024	1.80	2.83	3.11	4.96	0.05417	0.01920	0.03364	0.04358	0.05386	−336.45

**Figure A1:**
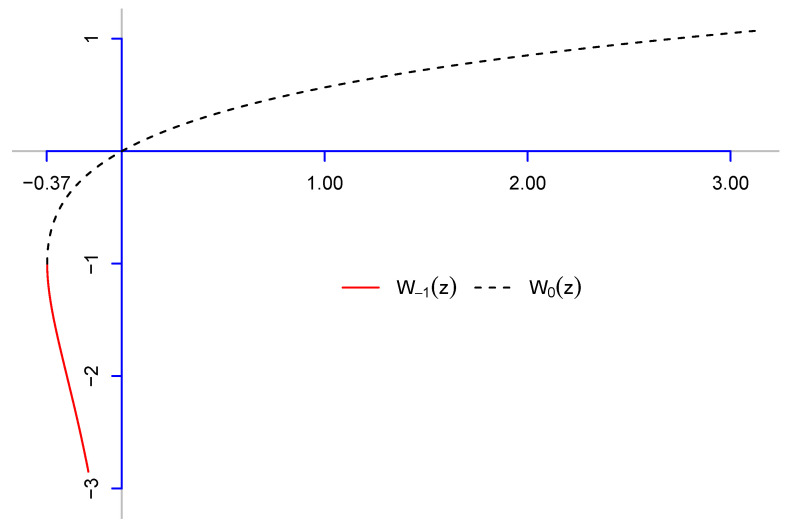
Lambert *W* function. $W_{-1}\left(z\right)$ denotes the lower branch and $W_{0}\left(z\right)$ denotes the upper branch.

## Appendix A. R Programs for the Proposed EM Algorithm

# Lindley.LS.EM function
Lindley.LS.EM <- function(Y, start = 1, maxits = 1000 L, eps = 1.0E-15) {
J = ncol(Y)
converged = logical(J)
para = numeric(J)
THETA = start
iter = numeric(J)
if( length(start) < J ) THETA = rep_len(start,J)
for ( j in 0L:(J-1L) ) {
thetaj = THETA[j+1L]
V = (J-j-1L) / (J-j)
y = Y[,j+1L]
while ( (iter[j+1L]<maxits)&&(!converged[j+1L]) ) {
w = mean(y+V*(thetaj+thetaj*y+2)/thetaj/(thetaj+thetaj*y+1))
newtheta = (1-w+sqrt( (w-1)^2+8*w ))/(2*w)
converged[j+1L] = abs(newtheta-thetaj) < eps
iter[j+1L] = iter[j+1L] + 1L
thetaj = newtheta
}
para[j+1L] = newtheta
}
list(para=para, iter=iter, conv=converged)
}
# Data
> Y0=c(21.02, 24.25, 6.55, 15.35, 39.08, 16.20, 34.59, 19.10, 28.22,
32.00, 11.25, 17.39, 28.47, 23.42, 42.06, 28.51, 34.56, 40.33,
27.56, 9.54, 27.09, 40.36, 41.44, 32.23, 7.53, 28.34, 26.32, 30.47)
> Y1=c(30.22, 45.54, 19.47, 16.37, 30.32, 4.16, 46.44, 38.40, 37.43,
45.52, 19.09, 25.43, 31.15, 31.28, 23.21, 33.59, 32.53, 15.35,
46.21, 36.21, 11.11, 33.21, 36.28, 8.17, 37.31, 35.58, 28.02, 40.4)
> Y2=c(43.43, 17.19, 23.28, 25.40, 43.53, 39.52, 16.33, 20.17, 25.41,
39.11, 11.59, 22.51, 2.41, 40.03, 45.36, 16.20, 40.44, 28.33,
28.05, 28.12, 23.33, 17.04, 19.13, 41.27, 13.43, 41.48, 29.33, 42.13)
Y = cbind(Y0, Y1, Y2)
# Use of Lindley.LS.EM() function
> Lindley.LS.EM(Y)
$para
[1] 0.03624714 0.04104211~0.06915810
$iter
[1] 58 37~2
$conv
[1] TRUE TRUE TRUE

## Appendix B. Generating Lindley-Distributed Random Numbers

The pdf of the Lindley distribution with the parameter θ>0 is defined as

$$f\left(x\right)=\frac{\theta^{2}}{1+\theta }\left(1+x\right)e^{-\theta x}=\left(\frac{\theta }{\theta +1}\right)\cdot \theta e^{-\theta x}+\left(1-\frac{\theta }{\theta +1}\right)\cdot \theta^{2}xe^{-\theta x}$$

for x>0. Thus, the Lindley distribution is regarded as the mixture of the exponential with rate θ and the gamma with (2,θ) with their mixing proportions being θ/(θ+1) and 1−θ/(θ+1) [35]. We can thus generate a Lindley random sample based on this mixture model. An alternative way is through the direct use of of the inverse cdf of the Lindley distribution, which depends on the Lambert *W* function. For the sake of completeness, we provide a brief introduction of the Lambert *W* function in the appendix and then show how it can be used to generate a Lindley random sample.

Lambert [37] first considered the Lambert’s transcendental equation which is the inverse of

(A1) $$z=W\left(z\right)e^{W(z)}.$$

The solution W(z) of the above equation was first recognized in [38].

Now we consider the cdf of Lindley in (6) and we let p=F(x) for notational convenience. Then we have

$$p=1-\frac{\theta +1+\theta x}{\theta +1}e^{-\theta x},$$

which leads to

(A2) $$-\left(\theta +1\right)\left(1-p\right)e^{-(\theta +1)}=-\left(\theta +1+\theta x\right)e^{-(\theta +1+\theta x)}.$$

If we let $z=-\left(\theta +1\right)\left(1-p\right)e^{-(\theta +1)}$, then we can rewrite (A2) as

(A3) $$z=-\left(\theta +1+\theta x\right)e^{-(\theta +1+\theta x)}.$$

Then it is immediate from (A1) and (A3) that

$$z=W\left(z\right)e^{W(z)},$$

where

(A4) W(z)=−(θ+1+θx).

Solving Equation (A4) for *x*, we obtain

(A5) $$x=\frac{-(\theta +1)-W(z)}{\theta }.$$

Substituting Equations (A3) into (A5), we have

(A6) $$x=\frac{-\left(\theta +1\right)-W\left(-\left(\theta +1\right)\left(1-p\right)e^{-(\theta +1)}\right)}{\theta }.$$

Please note that the Lambert function W(z) is not injective when *z* is in (−1/e,0) and has two branches as seen in Figure A1. The upper branch for the case of W>−1 is denoted by $W_{0}$ and the lower branch for the case of W<−1 is by $W_{-1}$. Then we can see that the *x* value in Equation (A6) is always positive in the lower branch and always negative in the upper branch. Thus, the quantile function or inverse cdf function of the Lindley distribution is explicitly expressed as

(A7) $$F^{-1}\left(p\right)=\frac{-\left(\theta +1\right)-W_{-1}\left(-\left(\theta +1\right)\left(1-p\right)e^{-(\theta +1)}\right)}{\theta }.$$

To ensure that the cdf possesses the standard properties of the cdf, we calculate the cdf values at the endpoints 0 and *∞* as follows. As p→1, we know $W_{-1}\left(0^{+}\right)\to -\infty$ and thus we have $F^{-1}\left(p\right)\to \infty$ as expected. When p=0, we have

$$F^{-1}\left(0\right)=\frac{-\left(\theta +1\right)-W_{-1}\left(-\left(\theta +1\right)e^{-(\theta +1)}\right)}{\theta }.$$

It is immediate from $W_{-1}\left(-ae^{-1}\right)=-a$ that $W_{-1}\left(-\left(\theta +1\right)e^{-(\theta +1)}\right)=-\left(\theta +1\right)$ and thus $F^{-1}\left(0\right)=0$ as expected. The Lambert *W* function is available in the lamW package [39] of the R language.
